# Mesenchymal stem cell-based therapies for treating well-studied neurological disorders: a systematic review

**DOI:** 10.3389/fmed.2024.1361723

**Published:** 2024-03-27

**Authors:** Gaurav Deepak Patel, Lichao Liu, Ailian Li, Yun-Hsuan Yang, Chia-Chi Shen, Beate Brand-Saberi, Xuesong Yang

**Affiliations:** ^1^Department of Anatomy and Molecular Embryology, Medical Faculty, Ruhr University Bochum, Bochum, Germany; ^2^Division of Histology and Embryology, International Joint Laboratory for Embryonic Development and Prenatal Medicine, Medical College, Jinan University, Guangzhou, China; ^3^School of Stomatology, Southwest Medical University, Guangzhou, China; ^4^School of Stomatology, Jinan University, Guangzhou, China; ^5^Clinical Research Center, Clifford Hospital, Guangzhou, China

**Keywords:** MSCs, neurological disorders, cell therapy, ALS, PD, MS, SCI, TBI

## Abstract

**Background:**

Millions of people across the globe are affected by conditions like Amyotrophic Lateral Sclerosis (ALS), Parkinson’s Disease (PD), Multiple Sclerosis (MS), Spinal Cord Injury (SCI), and Traumatic Brain Injury (TBI), although most occurrences are common in the elderly population. This systematic review aims to highlight the safety of the procedures, their tolerability, and efficacy of the available therapies conducted over the years using mesenchymal stem cells (MSCs) in treating the neurological conditions mentioned above.

**Methods:**

PubMed was used to search for published data from clinical trials performed using mesenchymal stem cells. Studies that provided the necessary information that mentioned the efficacy and adverse effects of the treatment in patients were considered for this review.

**Results:**

In total, 43 manuscripts were selected after a strategic search, and these studies have been included in this systematic review. Most included studies reported the safety of the procedures used and the treatment’s good tolerability, with mild adverse events such as fever, headache, mild pain at the injection site, or nausea being common. A few studies also reported death of some patients, attributed to the progression of the disease to severe stages before the treatment. Other severe events, such as respiratory or urinary infections reported in some studies, were not related to the treatment. Different parameters were used to evaluate the efficacy of the treatment based on the clinical condition of the patient.

**Conclusion:**

Mesenchymal stem cells transplantation has so far proven to be safe and tolerable in select studies and patient types. This systematic review includes the results from the 43 selected studies in terms of safety and tolerability of the procedures, and several adverse events and therapeutic benefits during the follow-up period after administration of MSCs.

## 1 Introduction

Cell-based therapies refer to the therapeutic injection of cellular material into individuals. These therapies utilize various cells, including stem cells, which are effective in treating degenerative diseases, blood cancers, and bone marrow disorders (1). Many published and ongoing clinical trials have investigated the use of cell therapy for neurological disorders such as Amyotrophic Lateral Sclerosis (ALS), Parkinson’s Disease (PD), Multiple Sclerosis (MS), Spinal Cord Injury (SCI), and Traumatic Brain Injury (TBI). These trials utilized cells obtained from either the patients themselves (autologous cells) or healthy donors’ bone marrow and peripheral blood (2). The use of autologous cells minimizes the risk of rejection associated with using donor cells. Mesenchymal Stem Cells (MSCs), non-hematopoietic stromal cells that primarily reside in the bone marrow but also occur in fat and other tissues, offer a promising therapeutic option for neurological disorders (3). Upon administration, MSCs migrate to the damaged neural tissue, secrete various cytotropic factors that intervene in the pathomechanisms of those diseases, provoke transdifferentiation of stem cells into neurons, and promote neurogenesis (4). Moreover, MSCs support hematopoiesis and assist in generating mesodermal lineage cells (5).

Alzheimer’s disease, also known as AD, is a neurodegenerative disorder that is the most common cause of dementia. Given AD’s multifaceted pathomechanisms, therapies focusing solely on a single aspect, such as amyloid-beta (Aβ) or tau, often fall short in demonstrating the efficacy of existing disease-modifying treatments (6). Consequently, adopting multi-target approaches could be more beneficial in managing this age-related neurodegenerative disorder than relying on single-target strategies (7).

For ALS, available treatments currently focus more on mitigating symptoms than on achieving a cure for the condition. ALS is a severe motor neuron disease that impairs the brain and spinal cord nerves responsible for movement, resulting in muscle weakness, shrinkage, and difficulties with speaking, swallowing, and breathing. While acute respiratory failure is uncommon, ALS’s severity is underscored by a mortality rate where 50% of patients pass away within 3 years of symptoms appearing; however, about 10% may survive beyond a decade (8). The Food and Drug Administration (FDA) has approved two drugs for ALS, Riluzole and Edaravone, for ALS management. These medications have demonstrated limited success in slowing disease progression and slightly prolonging patients’ lives (9).

Multiple sclerosis is a chronic inflammatory disease that affects the central nervous system (CNS), causing progressive damage to the myelin sheath that surrounds nerve fibers. This inflammation, persisting from months to years, results in a variety of debilitating symptoms such as motor, sensory, and cognitive impairments, which can culminate in disability, severe complications, and a significantly reduced quality of life (10). Current treatments for MS, including steroids, drugs for modifying the disease and drugs for targeting specific symptoms that may help in reduction of the frequency of exacerbations and slow the progression of the disease. However, there remains no effective strategy for repairing damage by regenerating myelin or neurons (11).

Current treatments for PD are primarily limited to managing symptoms without altering the disease’s progression. PD is a progressive neurodegenerative disease which occurs due to the degeneration of dopaminergic nigrostriatal neurons. Symptoms such as tremors, stiffness, slow movement, and imbalance characterize the early stages. As PD advances, other symptoms may also develop, such as motor impairment, dystonia, along with several other non-motor symptoms (12). Consequently, in the early phase, the rate of clinical decline is rapid, with a decrease of approximately eight to ten points on the Unified Parkinson Disease Rating Scale (UPDRS) in the first year (13).

Methylprednisolone is currently the only agent recognized for its effectiveness in treating SCI. Research has shown that it can reduce axonal damage caused by secondary injury processes (14). However, its use is constrained by a narrow treatment window of 8 h, modest effectiveness, and a significant risk of complications associated with high-dose corticosteroid therapy. SCI is a devastating neurological disorder. It may result in paralysis leading to functional deficit and clinical dependency (15). Currently available treatment strategies include drug therapy, surgical procedures, and rehabilitation training. Despite these advances, the improvements they provide are limited. Recent research has deepened our understanding of SCI’s molecular dynamics, yet discovering truly effective treatments continues to be difficult (16).

Recently, there has been a substantial improvement in the prehospital and intensive care facilities of patients with TBI, and there has also been development in the evidence-based guidelines for management of these facilities (17). TBI represents a significant global public health issue, notably leading to coma and disability among children and young adults (18). Severe TBI often results in disturbed consciousness and motor disorders, with prognosis remaining generally poor. Most patients show recovery within 6 months post-injury. However, a gradual improvement is possible over the following 12–18 months. The mortality rate for acute severe TBI is as high as 36%. Even under the best circumstances, 15% of patients will suffer severe disability, 20% will have moderate disability, and only 25% will make a complete recovery (19).

Adult-derived MSCs have been extensively studied in neurological diseases. These cells have several advantages over other stem cells, including ease of collection, high availability, and ease of culture; lower immunogenicity which allows the possibility of allotransplant, immunomodulation, no oncogenic transformation, and limited ethical concerns (20). The most dominant sources of MSCs are autologous or allogeneic bone marrow and adipose tissue, but they can also be isolated from other tissues, such as placenta, umbilical cord, and peripheral blood (21). Adipose tissues can be obtained by visceral or subcutaneous aspiration or excision of the fat tissue from the abdomen, femoral, brachium or gluteal areas of the patients (22). These methods include enzymatic digestion of the samples, red blood cell (RBC) removal with specific RBC lysis followed by cell filtration (23). Systemic injection of MSCs has been shown to have several limitations in clinical trials, including low cell survival and poor distribution in the CNS, but there are several encouraging trials as well (24). Alternative administration routes are being investigated to overcome these obstacles.

Due to the growing interest in stem cell applications for neurological disorders, this systematic review was performed to investigate safety, tolerability, efficacy, and related adverse effects of MSC therapies based on the data published in the literature.

## 2 Methods

This systematic review was conducted following the Preferred Reporting Items for Systematic Reviews and Meta-Analyses (PRISMA) guidelines (25).

### 2.1 Search strategy

The electronic database *PubMed* was used for this systematic review as it contains most of the published clinical trials related to the title of this systematic review. *Clinicaltrials.gov* was not used because most of the clinical trials were either ongoing, or still recruiting or were unpublished. The following keywords and combinations were used, and final selection was done on 30th of June 2023:

*(Neurodegenerative disorders OR Dementia OR Alzheimer’s Disease OR Vascular Dementia OR Lewy body Dementia OR Frontotemporal Dementia OR Huntington’s Disease OR Traumatic Brain Injury OR Creutzfeldt-Jakob Disease OR Parkinson’s Disease OR Spinal cord injury OR Multiple Sclerosis OR Amyotrophic Lateral Sclerosis)* AND *(Mesenchymal Stem Cells)* (The full search strategy is provided in Supplementary material 5).

### 2.2 Eligibility, inclusion and exclusion criteria

Experimental studies or clinical trials with information on the effects of mesenchymal stem cell therapies in patients with conditions like AD, ALS, PD, MS, SCI, and TBI met the eligibility criteria. The following records were eliminated from the selection process: books and documents, reviews, meta-analysis, systematic reviews, animal studies and other topics such as studies that did not use MSC therapies in the treatment of the chosen neurological conditions. After reading whole text of the selected articles, only focus on MSC therapies were included.

Reviewers (Xuesong Yang, Gaurav Patel, and Lichao Liu) independently assessed the records screened. After agreement, full text articles of every potentially eligible and relevant studies were downloaded. After reading whole text of the selected articles, only focus on mesenchymal stem cells therapies were included. Gaurav Patel and Lichao Liu extracted data from the selected studies and presented the extracted content in tables. This method allowed us to simultaneously screen eligible manuscripts and incorporate manuscripts that meet the criteria into this systematic review.

### 2.3 Data extraction

Information about the names of the authors, year of publication, country, type of study, follow-up period, characteristics of the participants, source of MSCs, administration route, improved condition, adverse events and clinical discussion were collected independently by the reviewers (Gaurav Patel and Lichao Liu) from the original reports. Our primary focus is on the improved condition of relevant diseases following MSC therapies, with the improved condition typically measured through corresponding assessment scales. For example, in the treatment of ALS, the improvement in patients after treatment is measured by the Revised ALS Functional Rating Scale. The evaluation of the condition of the patients, whether improvement or deterioration, was made using clinical indicators that were relevant to the conditions described in the selected studies; These parameters were chosen to measure the efficacy outcomes of these conditions. The patients in these clinical studies ranged in age from 18 to 80. For those studies that did not report relevant information, we assumed that they followed standard clinical practices in the industry.

### 2.4 Risk of bias assessment

We used the standard Cochrane Collaboration tool for evaluation of the risks of bias within the included studies (26). Based on this tool, the methodological quality of the included trials was evaluated and reviewed by two authors (Gaurav Patel and Lichao Liu).

## 3 Results

### 3.1 Design and samples

**FIGURE 1:**
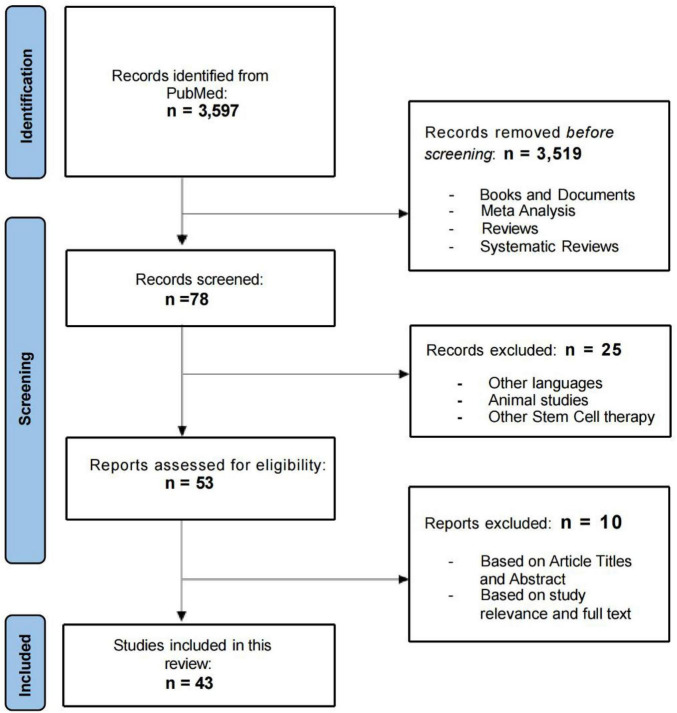
Flow chart of the included and excluded studies.

In this systematic review, the initial search of databases yielded 3,597 results. After the first screening, 3,519 results were excluded (books and documents, meta-analysis, reviews, and systematic reviews). After further evaluation of the titles and abstracts of the remaining 78 articles, we excluded 25 articles that were not published in English, did not involve MSC therapies, or were animal studies. The remaining 53 eligible articles were further analyzed by reviewers using standardized review forms. The research in these 43 articles involved the safety and/or efficacy of MSCs for the treatment of neurological diseases. Figure 1 shows the number of studies for each neurological conditions, included in this systematic review and the geographical distribution of the selected published studies. Few papers consisted of trials on multiple neurological conditions. Flow chart of the review process in presented in Figure 1.

### 3.2 Studies characteristics

43 studies were included in this systematic review, and their principal characteristics are presented in Table 1. All of the selected studies were clinical trials. The phases of these trials varied, with 30 being phase I, seven being phase II, and six being phase I/II. Although all the studies focused on the use of MSCs to treat neurological conditions, the sources of MSCs and the routes of administration varied. The sources of MSCs used in the 43 studies were as follows: 30 studies used autologous bone marrow-derived MSCs, five studies used autologous adipose-derived MSCs, six studies used autologous or allogeneic umbilical cord-derived MSCs, and one study used MSCs derived from placenta and peripheral blood. The routes of administration of MSCs varied. Six studies used intravenous (IV) infusion, 11 used intrathecal administration, three used both, and 13 studies injected MSCs into the spinal cord using lumbar puncture or direct injection at the injury site. Some studies also administered the cells via face and nasal cavity, intracerebroventricular route or cerebral arteries, and intraparenchymal, endovascular or intracisternal transplantation. The period of follow-up also varied, most of the studies followed-up for at least 6–12 months, with the shortest follow-up period being just 14 days and two studies had follow-up period of 7 and 9 years.

**TABLE 1:** Author, year and country of the published study, the type of disease studied and its design, source of MSC used for the study and its route of administration into the patients who participated in the study, number of patients and their characteristics like gender and age.

References, Country	Disease studied, study design	MSC source used for the study	Administration route
Mazzini et al. (35) Italy	Amyotrophic Lateral Sclerosis Phase I Clinical Trial	Autologous, Bone marrow derived	Injection into the spinal cord at different thoracic levels
Mazzini et al. (36) Italy	Amyotrophic Lateral Sclerosis Phase I Clinical Trial	Autologous, Bone marrow derived	Injection into the spinal cord at different thoracic levels
Mazzini et al. (37) Italy	Amyotrophic Lateral Sclerosis Phase I Clinical Trial	Autologous, Bone marrow derived	Intraparenchymal transplantation
Oh et al. (38) South Korea	Amyotrophic Lateral Sclerosis Phase I Clinical Trial	Autologous, Bone marrow derived	Intrathecal injection
Rushkevich et al. (33) Belarus	Amyotrophic Lateral Sclerosis Phase I Clinical Trial	Autologous, Bone marrow derived	Intravenous and Intra Lumbar injections
Staff et al. (39) USA	Amyotrophic Lateral Sclerosis Phase I Clinical Trial	Autologous, Adipose derived	Intrathecal injections
Syková et al. (40) Czech Republic	Amyotrophic Lateral Sclerosis Phase I/II a Clinical trial	Autologous, Bone marrow derived	Intrathecal injection
Siwek et al. (41) Poland	Amyotrophic Lateral Sclerosis	Autologous, Bone marrow derived	Intrathecal injection
Petrou et al. (42) Israel	Amyotrophic Lateral Sclerosis Phase II Clinical Trial	Autologous, Bone marrow derived	Intrathecal injection
Kim et al. (43) Republic of Korea	Alzheimer’s Disease	Umbilical cord blood derived	Intracerebroventricular injections
Brody et al. (44) USA	Alzheimer’s Disease	Lomecel-B MSC, Allogenic, Bone marrow derived	Intravenous infusion
Karussis et al. (4) Israel	Amyotrophic Lateral Sclerosis Multiple Sclerosis Phase I/II Clinical Trial	Autologous, Bone marrow derived	Intrathecal and Intravenous injections
Yamount et al. (31) USA	Multiple Sclerosis, Phase I Clinical Trial	Autologous, Bone marrow derived	Intrathecal and Intracisternal
Bonab et al. (11) Iran	Multiple Sclerosis Phase II Clinical Trial	Autologous, Bone marrow derived	Intrathecal injection
Connick et al. (45) UK	Multiple Sclerosis, Phase II a study	Autologous, Bone marrow derived	Intravenous infusion
Hou et al. (46) China	Multiple Sclerosis, Single Case Report	Allogenic, Umbilical cord derived, Autologous, Bone marrow derived	Intravenous infusion, Intrathecal infusion
Li et al. (47) China	Multiple Sclerosis, Phase II Clinical Trial	Umbilical cord derived	Intravenous infusion
Llufriu et al. (48) Spain	Multiple Sclerosis, Phase II Clinical Trial	Bone marrow derived	Intravenous injection
Lublin et al. (49) USA and Canada	Multiple Sclerosis Phase I study	Placenta derived, PDA-001	Intravenous infusion
Harris et al. (50) USA	Multiple Sclerosis, Phase I Clinical trial	Autologous, Bone marrow derived neural progenitors	Intrathecal injection
Fernández et al. (32) Spain	Multiple Sclerosis, Phase I/II Clinical Trial	Autologous, Adipose derived	Intravenous injection
Harris et al. (34) USA	Multiple Sclerosis, Phase I Clinical trial	Autologous, Bone marrow derived neural progenitors	Intrathecal injection
Riordan et al. (51) Panama	Multiple Sclerosis, Phase I/II Study	Umbilical cord derived	Intravenous injection
Petrou et al. (30) Israel	Multiple Sclerosis, Phase II Clinical Trial	Autologous, Bone marrow derived	Intrathecal or Intravenous injection
Venkataramana et al. (52) India	Advanced Parkinson’s Disease	Autologous, Bone marrow derived	Intracerebral transplantation
Canesi et al. (53) Italy	Progressive Supranuclear Palsy (PSP) – a form of PD	Bone marrow derived	Infusion into cerebral arteries
Carstens et al. (54) USA	Parkinson’s Disease	Autologous, Adipose derived	Face and Nasal cavity
Moviglia et al. (55) Argentina	Spinal Cord Injury, Therapeutic Protocol	Autologous, Bone marrow derived Transdifferentiated Neural Stem cells	Selective endovascular cell implant
Pal et al. (56) India	Spinal Cord Injury, Pilot Clinical Study	Autologous, Bone marrow derived	Lumbar puncture
Kishk et al. (28) Egypt	Spinal Cord Injury, Clinical Study	Autologous, Bone marrow derived	Intrathecal injection
Karamouzian et al. (57) Iran	Spinal Cord Injury Phase I/II Clinical Trial	Autologous, Bone marrow derived	Lumbar puncture
Dai et al. (58) China	Spinal Cord Injury Clinical Trial	Autologous, Bone marrow derived	Injection to the site of injury
Cheng et al. (59) China	Spinal Cord Injury	Umbilical cord derived	Lumbar puncture
El-Kheir et al. (29) Egypt	Spinal Cord Injury Phase I/II Clinical Trial	Autologous, Bone marrow derived	Lumbar puncture
Mendonça et al. (60) Brazil	Spinal Cord Injury Phase I Clinical Trial	Autologous, Bone marrow derived	Lumbar puncture
Oh et al. (38) South Korea	Spinal Cord Injury Phase III Clinical Trial	Autologous, Bone marrow derived	Injection to the site of injury
Hur et al. (61) Korea	Spinal Cord Injury Phase I Clinical Trial	Autologous, Adipose derived	Intrathecal injection
Satti et al. (62) Pakistan	Spinal Cord Injury Phase I Clinical Trial	Autologous, Bone marrow derived	Lumbar puncture
Vaquero et al. (63) Spain	Spinal Cord Injury Phase II Clinical Trial	Peripheral Blood derived	Intrathecal injection
Zhang et al. (64) China	Traumatic Brain Injury	Autologous, Bone marrow derived	Direct application, Intravenous infusion
Tian et al. (27) China	Traumatic Brain Injury	Autologous, Bone marrow derived	Lumbar puncture
Wang et al. (65) China	Traumatic Brain Injury	Umbilical cord derived	Lumbar puncture
Duma et al. (66) USA	Amyotrophic Lateral Sclerosis, Alzheimer’s Disease, Multiple Sclerosis, Parkinson’s Disease, Spinal Cord Injury, Traumatic Brain Injury	Autologous, Adipose derived	Intracerebroventricular injections

### 3.3 Main outcomes

**FIGURE 2:**
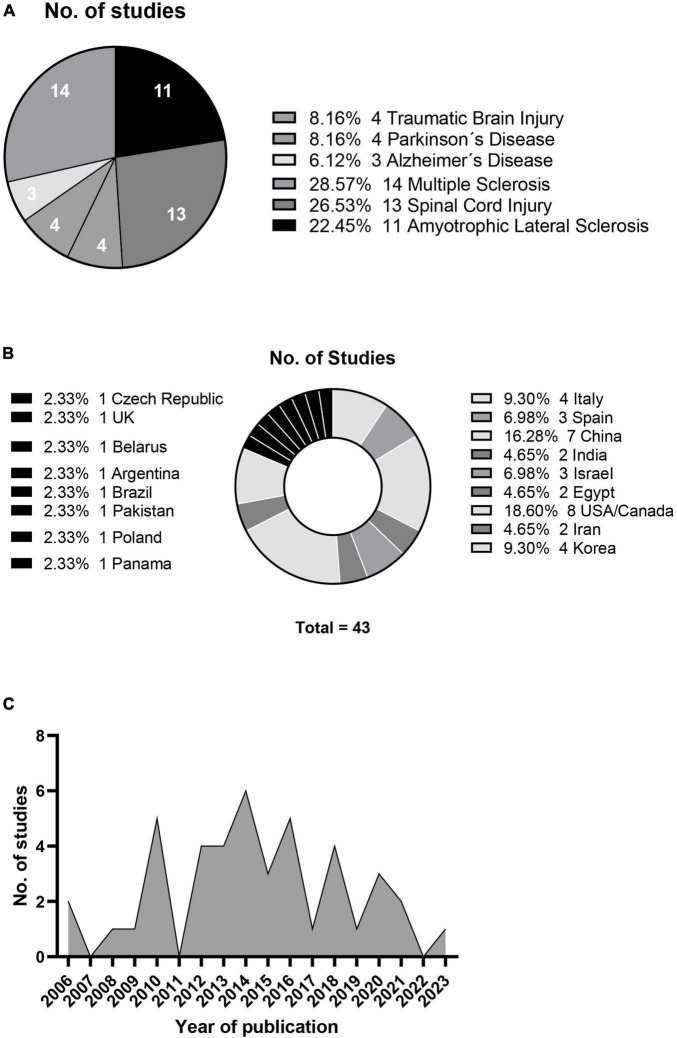
Neurological condition studies **(A)**, geographical distribution **(B)** and annual publication count **(C)**.

The relevant information statistics of the research papers included in this systematic review are shown in Figure 2, which categorizes them by disease type (A), country of origin (B), and publication year (C). The studies involved different types of disease, sources of mesenchymal stem cells, and patient numbers and ages. This review hence focused on two primary outcomes: adverse reactions after treatment and changes in clinical parameters for evaluating treatment efficacy for specific diseases.

In the studies that reported adverse events, eight studies reported frequent and low-grade fevers after treatment, regardless of the route of administration. Seven studies reported urinary or respiratory infections which were mild and tolerated by patients. Other common adverse effects reported were back pain or pain at the site of injection, which subsided in few hours or days. These common adverse effects were reported in 15 of the included studies while seven studies reported no adverse effects.

Three studies showed 100% improvement rate, with all their patients showing clinical improvements, but the sample size for those studies were relatively low, 10 or below. This is the reason why these studies requested for larger sample size for better evaluation results. Two of these studies had only two participants for PD and SCI. Thirteen other studies had improvement rate higher than or equal to 50%, but the limitation remained the same, of low sample size. One study on MS had only one participant whose improvement based on any clinical parameter, or score was not mentioned in the manuscript.

None of the studies had higher sample size, The highest sample size was 97 for a study related to TBI (27), 64 and 70 for SCI (28, 29) and 48 for MS (30), with improvement rates being 39%, 41% and 46% and 48% respectively.

Eight studies out of 43 included in this review showed no improvement, and 13 studies had no mention of any improvement scores due to their focus only on the safe administration of the cells and tolerance by the patients. The above results are summarized in Table 2.

**TABLE 2:** Author, year and country of the published study, the type of disease studied and its design, period of follow-up of the participants in the study, the percentage of improved patients, the adverse events that occurred in patients after treatment during the study as observed and reported during the follow-up period and the clinical discussions of the respective study.

References, Country	Disease studied, study design	Period of follow-up (months)	No. of patients with improved condition (percentage)	Adverse events	Clinical discussion
Mazzini et al. (35) Italy	Amyotrophic Lateral Sclerosis Phase I Clinical Trial	36	5 (71.42%)	2 patients died 9 months and 2 years after allogeneic bone marrow mesenchymal stem cell transplantation, respectively. 1 patient underwent tracheostomy for respiratory complications due to gastrointestinal pneumonia.	1. After MSC transplantation, a linear decline of the FVC and the ALSFRS was measured, well tolerated in 5 patients. 2. Long-term follow-up data confirmed the safety of autologous bone marrow mesenchymal stem cell expansion and spinal cord transplantation.
Mazzini et al. (36) Italy	Amyotrophic Lateral Sclerosis Phase I Clinical Trial	24	-	No deaths, or any adverse events, or toxicity problems related to MSC administration were reported.	1. No serious surgical complications were reported. Technical improvements were made to minimize the possible side effects and to promote transplantation of BM-MSCs to the affected regions of the spinal cord.
Mazzini et al. (37) Italy	Amyotrophic Lateral Sclerosis Phase I Clinical Trial	108	6 (31.57%)	The procedure was well tolerated by all patients. Mild and transient side effects related to surgery were reported.	1. After transplantation, 6 patients showed slowing down of disease progression.
Oh et al. (38) South Korea	Amyotrophic Lateral Sclerosis Phase I Clinical Trial	6, 12	-	12 patients showed musculoskeletal and connective tissue disorder. 4 patients reported back pain and 3 patients reported pyrexia. Pyrexia, pain and headache were not serious, but transient, which occurred during 4 days post treatment.	1. No improvement was reported in the decline of ALSFRS-R score, AALS score, or the FVC. 2. Despite lack of appropriate measures to detect efficacy of the treatment, The decline in ALSFRS-R scores during the 6-month follow-up period was more gradual than during the lead-in period, with scores remaining stable for the first 6 months after MSC injection.
Rushkevich et al. (33) Belarus	Amyotrophic Lateral Sclerosis Phase I Clinical Trial	12	-	1 patient developed fever. 2 patients developed post-dural puncture headache on the second day after treatment, with postural headache occurring when they attempted to stand up and return to a horizontal position.	1. In some patients, the safety of transplantation of cells and the significant improvement of the linear decline in FVC and functional status were noted. 2. The study concluded safety of autologous transplantation of cells into CNS but no convincing improvement in these patients were reported.
Staff et al. (39) USA	Amyotrophic Lateral Sclerosis Phase I Clinical Trial	1 - 27	-	Adverse events were observed in a dose-dependent fashion. Events included headache, low back and leg pain.	1. 17 participants reported improvements in bulbar function, limb strength, fasciculations, stiffness, and energy. These improvements were mild and temporary, but they were significant for the patients.2. All treated patients showed continued progression of disease based on longitudinal ALSFRS-R questionnaires.
Syková et al. (40) Czech Republic	Amyotrophic Lateral Sclerosis Phase I/II a Clinical trial	3, 6, 9, 12, 18	12 (52.17%)	Mild adverse events such as headache, hyperhidrosis, leukocytosis, and cystitis were observed. 1 patient died 2 months post administration of cells due to failure of respiratory system. Another patient experienced severe cervical stenosis with myelopathy and had to undergo surgery.	1. AE and MRI evaluation revealed safety of the procedure. 2. Beneficial effect of MSC was reported by some clinical findings on disease progression in some patients. 3. ALSFRS decline was reduced at 3 months after application, and the reduction persisted for 6 months in some cases. 4. More than 75% patients showed stable FVC and WS values at 3 months.
Siwek et al. (41) Poland	Amyotrophic Lateral Sclerosis	6	2 (25%)	1 patient had post-dural puncture headache.	1. The Wilcoxon test resulted in no statistically significant difference in the rate of progression before, during, and after treatment. 2. Rate of progression decreased in 2 patients, increased in 2 patients, and remain unchanged in the other 4 patients.
Petrou et al. (42) Israel	Amyotrophic Lateral Sclerosis Phase II Clinical Trial	24	19 (95%)	3 patients experienced headache and back pain. 1 patient reported general weakness and fatigue. All side effects were mild. 1 patient reported rapid disease deterioration and failure of respiratory system, deceased 4 weeks after first treatment.	1. Statistically significant difference was observed when post and pretreatment periods were compared based on the Kaplan-Mayer survival curve that shows ALSFRS-R progression incidence. 2. 25% slower rate of progression was observed after treatment in ALSFRS-R.
Kim et al. (43) Republic of Korea	Alzheimer’s disease	18, 24, 36	All 9 (100%)	9 patients experienced fever. 7 patients experienced headache. 4 patients experienced vomiting. Low-dose patients with fever, nausea, and vomiting required an additional day of hospitalization. No dose-limiting toxicities were observed.	1. Clinical efficacy of hUCB-MSCs injection could not be proved by this study. 2. CSF samples collected 1 day after injections showed reduction in the levels of total tau, phosphorylated tau, and Aβ42. 3. Increased levels 4 weeks after transplantation suggests inefficiency of the method. The short lifespan of the cells may have limited the duration of the therapeutic effects.
Brody et al. (44) USA	Alzheimer’s disease	13	-	1 patient died at day 144 in the high dose Lomecel-B arm. No other adverse events were deemed related to the study product.	1. Consistent with the efficacy assessments like MMSE, ADAS-Cog-11, and QOL-AD results, TMT-A, TMT-B and GDS reported improvements over placebo, but these improvements were not significant.
Karussis et al. (4) Israel	Multiple sclerosis Amyotrophic lateral sclerosis Phase I/II clinical trial	25	MS: 11 (73.33%)	21 patients reported adverse effects due to injection, consisting of transient fever. 15 patients reported headache.	1. MSCs are clinically feasible and comparatively safer procedure for administration. It has been shown to have immediate immunomodulatory effects. 2. The mean ALSFRS score didn’t change during the observation period of first 6 months, but improvement in the mean EDSS score was observed from 6.7 to 5.9.
Yamount et al. (31) USA	Multiple sclerosis, Phase I clinical trial	3, 6 and 12	6 (85.71%)	1 patient reported seizures and transient encephalopathy a few days after cell injection. Another patient reported transient cervical and low back pain for a few days, without fever or meningeal signs.	1. 6 patients improved on different components of EDSS and MSFC post 3 – 6 months of treatment. 2. For longer follow-up, 4 patients maintained overall clinical improvement at 1 year. 3. Despite clinical improvement, MRI showed evidence of disease progression.
Bonab et al. (11) Iran	Multiple sclerosis Phase II clinical trial	12	4 (16%)	Transient low-grade fever, headache, nausea/vomiting were some short-term adverse events associated with injection along with weakness in the lower limbs. No other severe adverse effect was reported.	1. Based on the data in 1 year post injection, MSC therapy can potentially improve the course of disease with some mild or transient adverse effects. 2. Mean EDSS of 22 patients changed from 6.1 to 6.3. The numbers improved in 4 patients, showed deterioration in 6 and showed no change in 12 patients. 3. Absence of a control group was a limitation.
Connick et al. (45) UK	Multiple sclerosis, Phase II a study	10	All 10 (100%)	A transient rash was observed in 1 patient shortly after treatment. 2 patients developed self-limited bacterial infections 3–4 weeks after treatment.	1. Procedure is feasible and safe. 2. Consistent with neuroprotection, the study also suggests structural, functional, and physiological improvement after treatment.
Hou et al. (46) China	Multiple sclerosis, Single case report	48	-	Following the infusion, some patients noted fever, headache, rash, and dizziness. No significant adverse events were reported. The treatment was well tolerated by the patients.	1. Allogenic hUC-MSC can be obtained in large numbers and conveniently than BM-MSC. 2. The systemic infusion of a large number of non-HLA matched hUC-MSCs is likely to be safe without significant graft-versus-host disease. 3. Improvement in EDSS values from 3.5 to 2.0 could be because of natural recovery associated with Natalizumab over the period of treatment. 4. Based on *in vitro* data, the secreted trophic factor can promote repair.
Li et al. (47) China	Multiple sclerosis, Phase II clinical trial	12	-	No significant adverse effect was reported during 1-year observation period.	1. Patients from treatment group had a steadier disease course, incidences of relapse were few in number than the control group who received only anti-inflammatory and immunosuppressive treatment. 2. EDSS scores showed slight improvement at the end of 1-year period, lower than that of the baseline scores.
Llufriu et al. (48) Spain	Multiple sclerosis, Phase II clinical trial	6, 12	2 (22.22%)	1 patient had a facial flushing during infusion. Other events included upper respiratory infections in four, three gastroenteritis, one dental abscess, one patient tested positive for influenza virus infection, all of them graded as mild.	1. At 6 months and at the end of the study, EDSS or MSFC z-score didn’t show any significant difference. 2. The number of participants in the study was limited. Lack of beneficial effects could be due to the crossover design of the study.
Lublin et al. (49) USA and Canada	Multiple sclerosis Phase I study	6, 12	10 (62.5%)	Reactions like headache, upper respiratory infection, nausea, fatigue, disturbance in the gait, infection of the urinary tract and nasopharyngitis. Couple of patients from the low-dose group and 4 patients from the high-dose group reported swelling at the site of infusion, hematoma, mass, or pain. 2 patients in the high-dose group also reported anaphylactoid reaction and superficial thrombophlebitis.	1. Similar to baseline or improved EDSS scores were reported for majority of patients over the follow-up period of 1-year. 2. Infusion of PDA-001 (MSC-like cells) is feasible and safe. 3. Possible mechanism for the action of PDA-001 in MS was not mentioned.
Harris et al. (50) USA	Multiple sclerosis, Phase I clinical trial	7.4 years	4 (66.66%)	The patients experienced mild fever amd mild headache for one and four times, respectively. One patient experienced moderate headache that lasted for a week.	1. 4 patients showed improved EDSS of 0.5 to 1.0. 2. 2 patients reported improved functioning of bladder who experienced bladder dysfunction. 3. 1 patient reported improvement in bowel function after treatment. 4. During treatment, none of the patients reported deterioration in any aspect of the disease.
Fernández et al. (32) Spain	Multiple sclerosis, Phase I/II clinical trial	12	-	2 deaths occurred during the study. 1 patient reported choking and bronchial aspiration. Other suffered respiratory infection. Other frequent adverse events included urinary infection and anemia.	1. Mean EDSS score showed no statistically significant difference over the course of the study. 2. Individual EDSS changes were not significant as well.
Harris et al. (34) USA	Multiple sclerosis, Phase I clinical trial	6	8 (40%)	Transient headaches and fever were the most common minor adverse events within 24 h of treatment.	1. 8 patients showed 0.5-point improvement in EDSS, 4 of which improved by 2.0 or better compared to baseline. 2. The EDSS of 10 patients remained stable throughout the study. 3. 2 patients showed decline in their EDSS score.
Riordan et al. (51) Panama	Multiple sclerosis, Phase I/II study	12	-	18 patients experienced mild headache, 1 had moderate headache. 19 patients had mild fatigue.	1. After a month post treatment, the mean EDSS decreased to 4.75 (mean reduction of about 1 category). EDSS further improved to 4.62 post 1-year. 2. The bladder/bowel/sex dysfunction category showed statistically significant improvement at 1-month from the baseline assessment.
Petrou et al. (30) Israel	Multiple sclerosis, Phase II clinical trial	12	23 (47.91%)	Headache, back pain, and cervical pain were the most common adverse events.	1. MSCs treatment group showed significant improvement in ambulation index, sum of functional scores, 25-foot timed walking test, 9-hole peg test, PASAT, and OWAT/KAVW cognitive tests, as well as the rate of change in T2 lesion load on MRI, and in newer biomarkers such as OCT (retinal nerve fiber layer) and functional MRI.
Venkataramana et al. (52) India	Advanced Parkinson’s disease	10 - 36	3 (42.85%)	No serious adverse events were reported after transplantation of stem cells.	1. 3 patients reported steady improvement in their off/on UPDRS. Mean off score improved by 22.9% from baseline, whereas mean on score improvement was 38%. 2. H&Y and S&E showed similar improvements. 3. Protocol seems to be safe.
Canesi et al. (53) Italy	Progressive Supranuclear Palsy (PSP) – a form of Parkinson’s disease	3, 6, 12	4 (80%)	1 patient developed transient left hemiparesis. MRI of the brain performed 24 h after administration of the cells showed ischemic alterations in the posterior segment of the left inferior peduncle of the cerebellum and in the right mesencephalon.	1. All patients showed stable clinical scores on at least two validated scales at the last follow-up study. 2. 1 patient maintained stable clinical scores for at least 1 year. 3. Although not applicable to patients with severe impairment, biomechanical evaluation has been shown to be a reliable method for investigating motor and postural capabilities in PSP patients.
Carstens et al. (54) USA	Parkinson’s disease	P 1 - 60 P 2 - 12	Both (100%)	No adverse events have been mentioned.	1. Since there were only 2 subjects, it is difficult to firmly conclude from these data and is therefore an important methodological drawback. 2. Both patients reported qualitative improvements in motor and nonmotor symptoms following cell injection.
Moviglia et al. (55) Argentina	Spinal cord injury, Therapeutic protocol	P1: 2, 3 P2: 2	Both (100%)	No adverse events were detected.	1. The potential risk of neoplastic transformation was eliminated with the use of adult NSC in this study. 2. The risks of graft rejection and the need for immunosuppressant drugs was minimized by the use of autologous cells which also minimized the possible risks and problems that could arise with donor selection. 3. The risks of neurosurgery or intradural injection was eliminated by minimally invasive and intravascular procedures.
Pal et al. (56) India	Spinal cord injury, Pilot clinical study	36 – 3P 24 – 10P 12 – 10P	20 (66.67%)	6 deaths were reported during the follow-up period. There were no serious adverse events related to the treatment. Sensory and motor functioning remained unchanged after stem cell administration.	1. Autologous transplantation of cells into lumbar space through intrathecal route is safe, feasible and beneficial. 2. Significant improvement in the daily activities of the patients reported. Improved quality of life, including restoration of bladder and bowel sensation was also reported. 3. Patients suffering from thoracic or cervical injury for over 6 months showed no improvement.
Kishk et al. (28) Egypt	Spinal cord injury, clinical study	12	18 (40.90%)	1–2 days after each injection, 24 patients reported neuropathic pain below the level of their lesion. After 4 injections, 3 patients reported excessive sweating below the level of the lesion. This continued throughout the duration of follow-up. After MSC injection, 2 patients reported transient hypertension for 2 days. Having the history of post-infectious myelitis, 1 patient experienced encephalomyelitis after her third injection.	1. No significant changes were observed in baseline measures. No differences between the MSC group and control group were reported. 2. Improvement in the motor scores increased by 1–2 points in a higher percentage of MSC group. Changed ASIA A to B. The groups showed no significant improvements in clinical measures. 3. Patients with a history of myelitis may experience side effects by the use of autologous MSCs.
Karamouzian et al. (57) Iran	Spinal cord injury Phase I/II clinical trial	12 - 33	5 (45.45%)	No clinical adverse effects of cell therapy. Not significant, but more frequent pain was reported in 73% of the study group.	1. Improvement in neurological function was reported at subacute stage in 45.5% of the patients after cell transplantation. 2. ASIA A to ASIA C, a two-grade improvement from baseline was marked in patients. 3. Small number of patients and their heterogeneity was a problem for reliable analysis of the efficacy despite differences in the recovery levels between the groups.
Dai et al. (58) China	Spinal cord injury Clinical trial	6	10 (50%)	Transient adverse reactions like fever, headache and dizziness were reported by some patients after transplantation. Severe nausea and vomiting were reported post the adverse reactions. Meningeal irritation signs were negative. MRI results showed no signs of tumor until 6 months after transplantation of the cells.	1. Motor function, ASIA score and EMG data showed significant improvement in the treatment group. 2. Between the treatment group and the control group, significant differences were observed in sensory function, ASIA score, and PSSEP data.
Cheng et al. (59) China	Spinal cord injury	6	12 (50%)	Radiating neuralgia was reported in only 1 patient from the treatment group.	1. Based on scaled ratings and urodynamic examinations, UC-MSC transplantation showed advantages in neurofunctional recovery compared to rehabilitation therapy and control group. 2. This method has been proven to alleviate tension in the lower limb muscle, increase strength of the limb, and improve urinating functions.
El-Kheir et al. (29) Egypt	Spinal cord injury Phase I/II clinical trial	18	23 (46%) Treatment Group	After the cell injection, mild side effects like headache and pain are reported at the site of puncture immediately after the procedure. 15 patients suffered from neuropathic pain post infusion. No long-term side effects were detected.	1. From AIS B to AIS C, 17 patients from treatment group showed improvement. AIS conversion from AIS A to AIS B or C. 2. None from the control group participants showed AIS conversion or improvement. 3. Patients from treatment group reported improvement in neurological functions as early as 4–6 weeks post-transplant.
Mendonça et al. (60) Brazil	Spinal cord injury Phase I clinical trial	3, 6	7 (50%)	Low-intensity pain at the site of incision was the most frequent symptom post operation. 1 patient developed a postoperative complication, evolving cerebrospinal fluid leak. None had fever, infection, or meningitis.	1. Autologous MSC intralesional transplantation is a safe, feasible, and potentially neurologically beneficial procedure for patients. 2. Statistically significant improvement in ASIA motor scores from B to C was reported after 6 months of transplantation.
Hur et al. (61) Korea	Spinal cord injury Phase I clinical trial	8	5 (35.71 %)	3 patients reported adverse events like infection of the urinary tract, headache, nausea, and vomiting.	1. 5 patients showed improvement in ASIA motor score. 2. 10 patients showed improvement in sensory function but 1 showed sensory deterioration. 3. Some patients showed mild clinical effectiveness in this pilot study, regardless of uncertain radiological and electrophysiological results.
Satti et al. (62) Pakistan	Spinal cord injury Phase I clinical trial	12, 24	-	1 patient reported headache. Nonspecific tingling sensation was reported by 2 patients. No other serious side effects or complications were reported.	1. Small size of the participants and uncontrolled nature of the study are the few limiting factors in drawing any conclusions regarding the treatment efficacy.
Vaquero et al. (63) Spain	Spinal cord injury Phase II clinical trial	4, 7, 10	7 (63.63%)	4 patients reported mild transitory sciatic pain, headache and pain at the site of lumbar puncture.	1. 3 patients reported improvement. Initially classified as ASIA A, B and C changed to ASIA B, C and D, respectively in these 3 patients. 2. 66.6% patients improved in urodynamics studies. 3. 55.5% patients improved in neurophysiological studies. 4. 44.4% patients showed improvement in contraction of voluntary muscle.
Zhang et al. (64) China	Traumatic brain injury	6	All 7 (100%)	No deaths or serious adverse events related to treatment were reported. No immediate or delayed toxicity was reported related to administration of MSC.	1. Procedure proved feasible and safe. 2. Combined procedures of cell delivery enhanced the efficacy of MSC homing. 3. Significant improvement in neurologic severity was reported by the third month post treatment and further improvement was reported at the sixth month post cell therapy.
Tian et al. (27) China	Traumatic brain injury	14 days	38 (39.17%)	Symptoms like transient fever was reported by 56 patients. After cell therapy, 2 patients reported light headache on second day. No adverse events like inflammation, systemic infections or gastrointestinal bleeding were observed.	1. Possibly due to effects of media, number of cells or isolation methods, a large number of patients, 59 of 97 patients, who received stem cell therapy did not show any improvement, 2. Improvement in younger patients was reported to be higher in comparison to the older ones.
Wang et al. (65) China	Traumatic brain injury	6	-	Low intracranial pressure reactions like mild dizziness and headache were reported in 4 patients within 48 h of injection. These reactions were sometimes followed by nausea and vomiting. No obvious abnormalities were found in body temperatures, heart rates, blood pressures, oxygen saturations and respiratory rates of all the patients during the treatment.	1. The treatment group showed improvements in upper extremity motor sub-score, lower extremity motor sub-score, sensation sub-score, and balance sub-score on the FMA. 2. The FIM scores improved in all seven sub-scores, including patient self-care, sphincter control, mobility, locomotion, communication, and social cognition.
Duma et al. (66) USA	Amyotrophic lateral sclerosis, Alzheimer’s disease, multiple sclerosis, Parkinson’s disease, Spinal cord injury, traumatic brain injury	2 - 36	ALS: 0 AD: 5 (50%) MS:2 (33.33%) PD: 1 (16.67%) SCI: 1 (100%) TBI: 0	Other than transient meningismus mild headache, or pain at the site of surgery, no infections or complications were reported by the participants for less than 24 h	1. Of the 31 patients, 6 died. 2. Anti-inflammatory and immunomodulatory effects were reported in SVF. 3. Promising clinical improvement or stability was observed in the AD and MS groups as secondary endpoints. 4. Earlier intervention in ALS and PD groups may yield better results.

### 3.4 Risk of bias assessment

A summary of the risk of bias assessment for each study is shown in Supplementary material. When more than five of the seven assessment criteria are rated as low risk, the study is considered to have an overall low risk of bias. Therefore, studies classified with a low risk of bias include: Five on ALS (**S1**), one on AD (**S2**), six on MS (**S2**), two on PD (**S3**), five on SCI (**S3** and **S4**), and two on TBI (**S4**).

## 4 Discussion

This systematic review explored the increasing interest and significance of MSC therapies on some of the serious neurological conditions like AD, ALS, MS, PD, SCI and TBI that affect millions of people worldwide (1). MSCs are a promising stem cell type with the advantages of pluripotency, immunomodulatory properties, and low immunogenicity (3). This systematic review primarily investigated the safety of the procedures used in the studies, adverse effects after treatment, and efficacy of MSC therapy for the above-mentioned conditions.

Many of the included studies had limitations, such as small sample sizes and short follow-up periods. In certain cases, with only one or two participants, assessing the treatment’s efficacy proved challenging. There were some serious adverse effects and even deaths, most were not attributed to the treatment itself, as described by the authors. Minor adverse effects in most of the cases were either pain in the site of injection or headache or nausea, which were mild and subsided soon. These minor adverse events reported can be deemed negligible if the treatment resulted in better efficacy rate. Nonetheless, a significant concern remains regarding the potential for long-term adverse events, given the short follow-up periods in many studies.

Autologous stem cells, unlike allogeneic stem cells from donors, do not pose the risk of immunogenic reactions or transmission of other diseases. Notably, none of the studies mentioned in this systematic review reported any rejection of cells by the patients after treatment. The studies that used allogenic MSCs did not report any improvement in individual patients, but an overall improvement was reported. It was not clearly confirmed if the improvement shown was due to allogenic cells or natural recovery. Hence, further studies need to be performed to validate the efficacy of use of allogenic MSCs.

This systematic review primarily investigates the efficacy and safety of MSC therapies, but the optimal administration route for MSCs in patients is also worth discussing. Some studies have suggested that IV infusion may be as effective as other administration routes (30–32). Other studies have suggested that IV infusion may reduce the number of cells that can migrate to the CNS. Therefore, intrathecal administration may be a more efficacious route of delivery (33, 34). However, due to the limited number of studies using the intrathecal route and the small number of participants in those studies, encouraging this route will be difficult until a sufficient number of successful studies adequate sample size are recorded. Autologous bone marrow-derived MSCs (BM-MSCs) were the most prevalent type of cells in the selected studies. These cells can be harvested from adult bone marrow with ease and safety. There is considerable variation in patient ages and stages of disease progression among the studies, with specific details available in Tables 1, 2, making it impossible to generalize these outcomes to other patients. The heterogeneity of research methods used in different studies makes it challenging to draw definitive conclusions. Although the search strategy included two databases, *PubMed* and *Clinicaltrials.gov*, results from only *PubMed* were selected for further eligibility, inclusion and exclusion process because most of the clinical trials resulted from *Clinicaltrials.gov* search were either ongoing, or still recruiting or were unpublished. Inclusion of studies only from one database in this case could be a possible limitation of this systematic review.

## 5 Conclusion

This systematic review focused on the cell therapies based on transplantation of MSCs to patients suffering from deadly neurological disorders likes AD, ALS, MS, PD, SCI and TBI. MSCs transplantation is generally considered to be a safe and well-tolerated therapy, according to the findings of most studies included in this systematic review. However, stronger evidence in the results are still needed to know the exact efficacy and potency of this therapy. In order for these treatments to be considered for clinical practice and benefit millions of patients, future research should focus on larger-scale clinical trials and longer follow-up periods.

## Data availability statement

The original contributions presented in this study are included in the article/Supplementary material, further inquiries can be directed to the corresponding authors.

## Author contributions

GP: Conceptualization, Investigation, Writing – original draft. LL: Formal analysis, Visualization, Writing – original draft. C-CS: Methodology, Writing – original draft. AL: Data curation, Methodology, Writing – original draft. Y-HY: Data curation, Methodology, Writing – original draft. BB-S: Conceptualization, Supervision, Writing – review and editing. XY: Conceptualization, Methodology, Supervision, Writing – review and editing.
