# Supplementary material for: Mesenchymal stem cell-based therapies for treating well-studied neurological disorders: a systematic review
Source: Front Med (Lausanne). 2024 Mar 27;11:1361723. doi: 10.3389/fmed.2024.1361723 (PMC11004389; doi:10.3389/fmed.2024.1361723)
Supplement: Supplementary file 1 [file Data_Sheet_1.docx]

**Supplementary materials**

**S1. Risk of bias assessment for the included studies (ALS).**

**
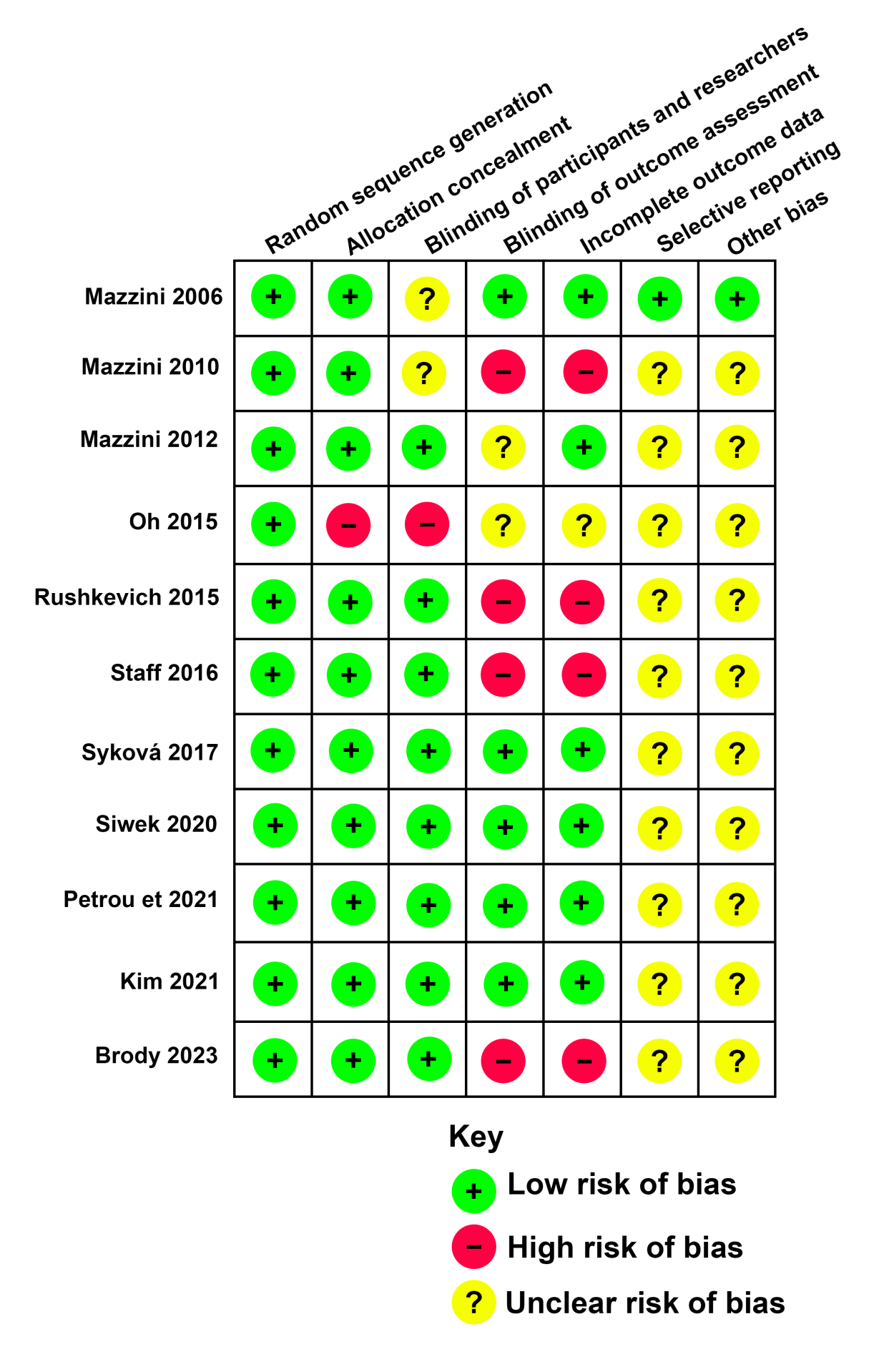
**

**S2. Risk of bias assessment for the included studies (AD and MS).**

**
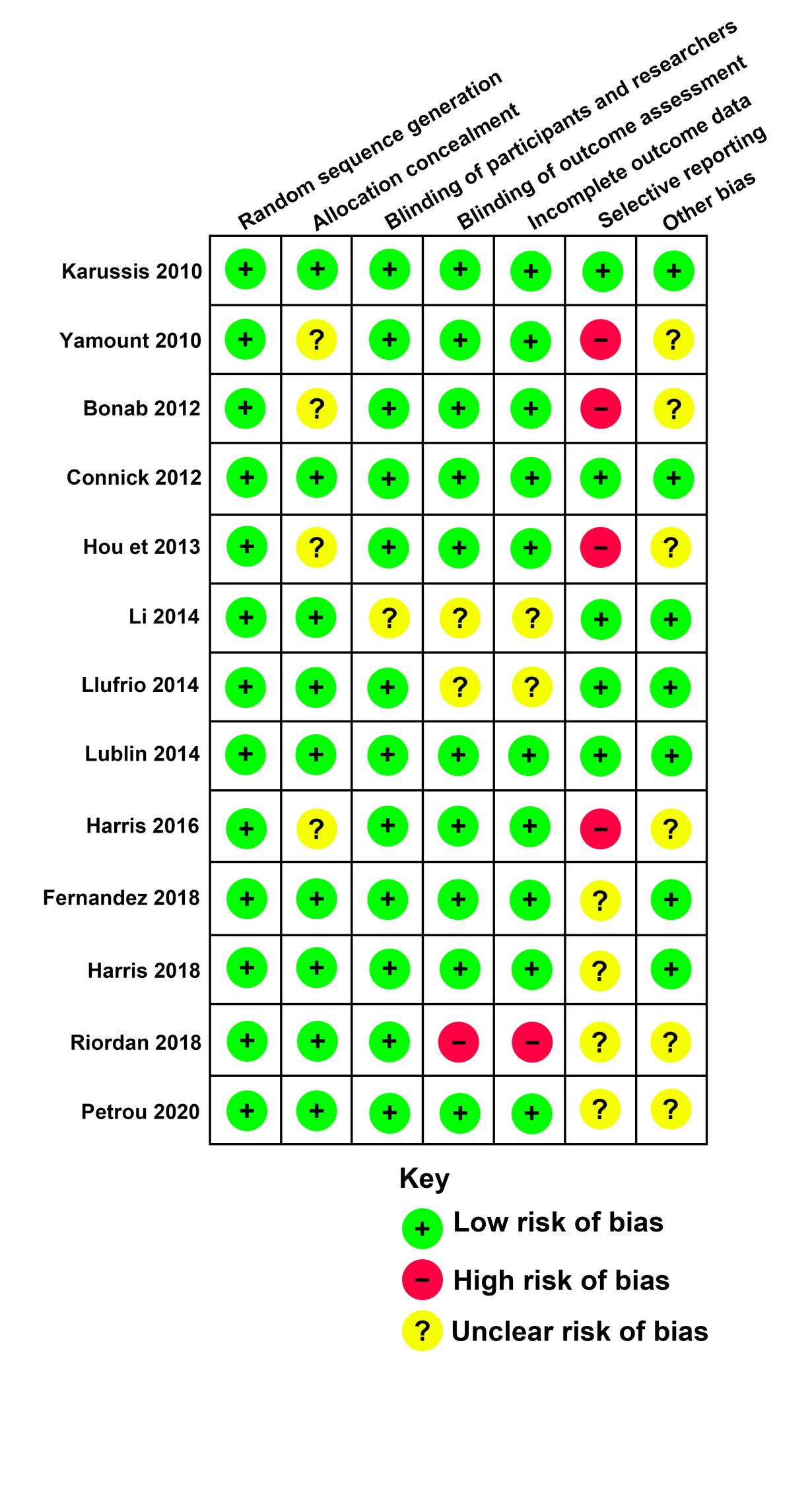
**

**S3. Risk of bias assessment for the included studies ( PD and SCI).**

**
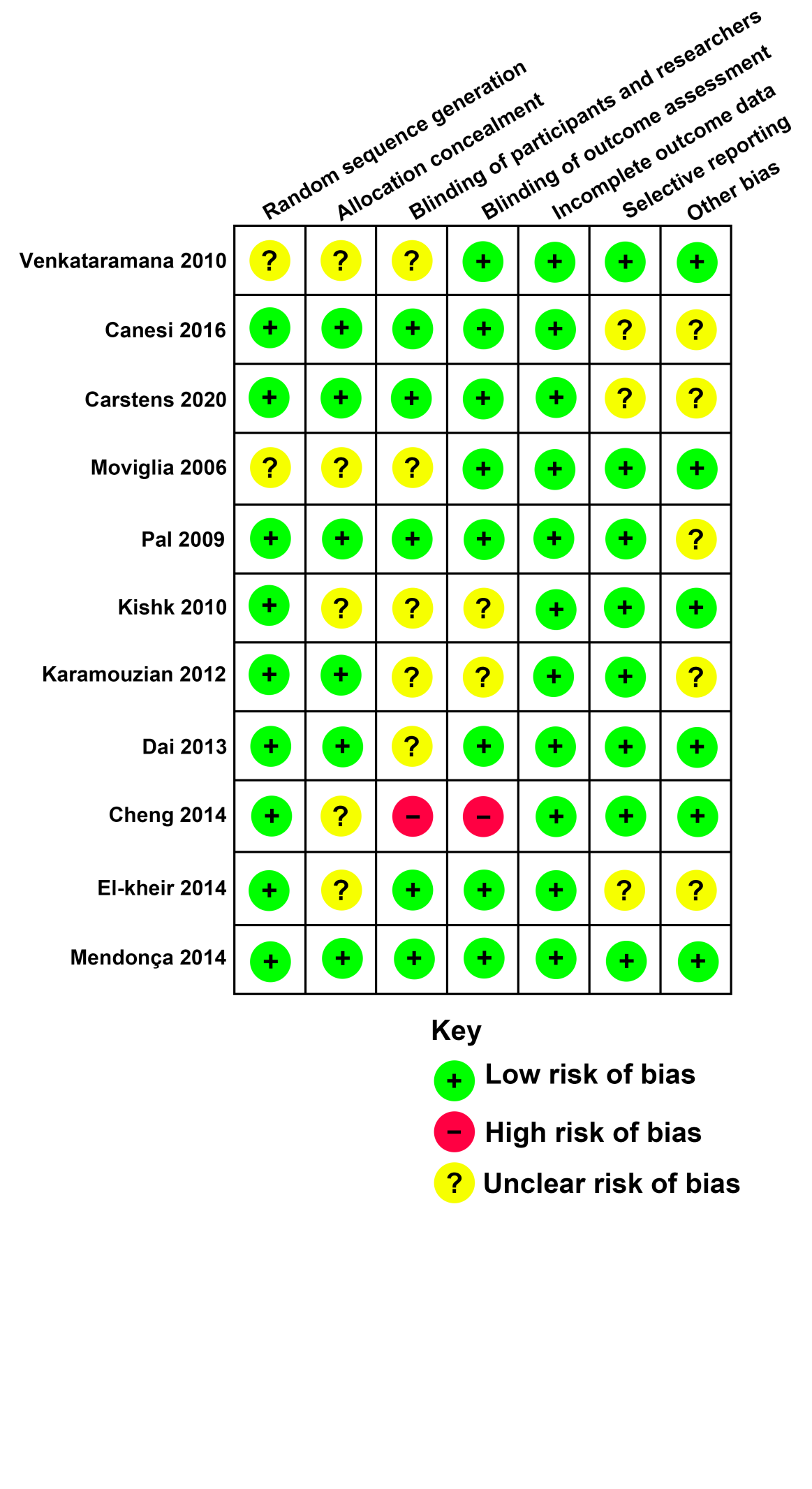
**

**S4. Risk of bias assessment for the included studies ( SCI and TBI).**

**
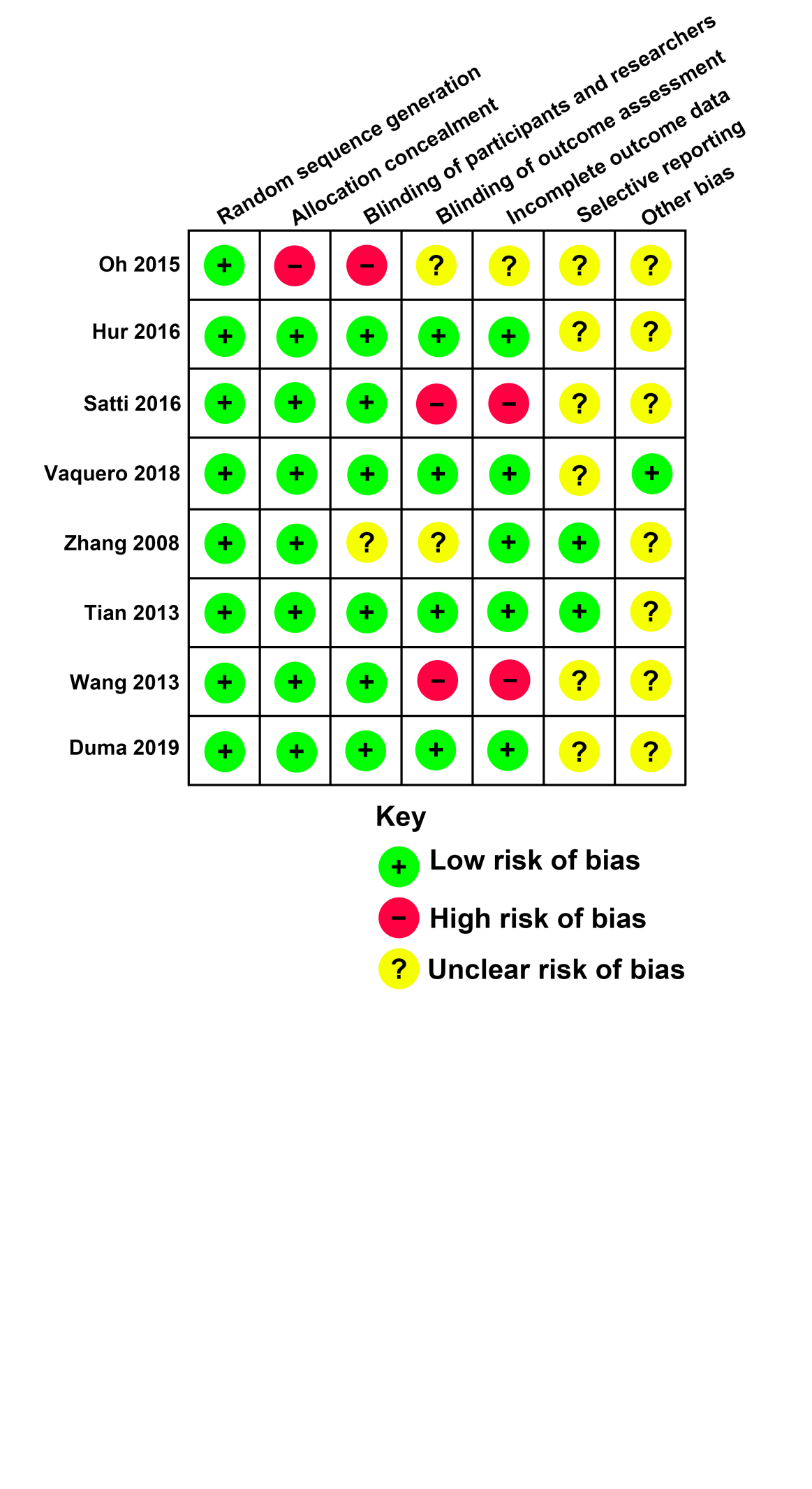
**

**S5. Search strategy**

**Search date: 30th of June 2023**

1. **PubMed**

("neurodegenerative diseases"[MeSH Terms] OR ("neurodegenerative"[All Fields] AND "diseases"[All Fields]) OR "neurodegenerative diseases"[All Fields] OR ("neurodegenerative"[All Fields] AND "disorders"[All Fields]) OR "neurodegenerative disorders"[All Fields] OR ("dementia"[MeSH Terms] OR "dementia"[All Fields] OR "dementias"[All Fields] OR "dementia s"[All Fields]) OR ("alzheimer disease"[MeSH Terms] OR ("alzheimer"[All Fields] AND "disease"[All Fields]) OR "alzheimer disease"[All Fields] OR ("alzheimers"[All Fields] AND "disease"[All Fields]) OR "alzheimers disease"[All Fields]) OR ("dementia, vascular"[MeSH Terms] OR ("dementia"[All Fields] AND "vascular"[All Fields]) OR "vascular dementia"[All Fields] OR ("vascular"[All Fields] AND "dementia"[All Fields])) OR ("lewy body disease"[MeSH Terms] OR ("lewy"[All Fields] AND "body"[All Fields] AND "disease"[All Fields]) OR "lewy body disease"[All Fields] OR ("lewy"[All Fields] AND "body"[All Fields] AND "dementia"[All Fields]) OR "lewy body dementia"[All Fields]) OR ("frontotemporal dementia"[MeSH Terms] OR ("frontotemporal"[All Fields] AND "dementia"[All Fields]) OR "frontotemporal dementia"[All Fields]) OR ("huntington disease"[MeSH Terms] OR ("huntington"[All Fields] AND "disease"[All Fields]) OR "huntington disease"[All Fields] OR ("huntingtons"[All Fields] AND "disease"[All Fields]) OR "huntingtons disease"[All Fields]) OR ("brain injuries, traumatic"[MeSH Terms] OR ("brain"[All Fields] AND "injuries"[All Fields] AND "traumatic"[All Fields]) OR "traumatic brain injuries"[All Fields] OR ("traumatic"[All Fields] AND "brain"[All Fields] AND "injury"[All Fields]) OR "traumatic brain injury"[All Fields]) OR ("creutzfeldt jakob syndrome"[MeSH Terms] OR ("creutzfeldt jakob"[All Fields] AND "syndrome"[All Fields]) OR "creutzfeldt jakob syndrome"[All Fields] OR ("creutzfeldt"[All Fields] AND "jakob"[All Fields] AND "disease"[All Fields]) OR "creutzfeldt jakob disease"[All Fields]) OR ("parkinson disease"[MeSH Terms] OR ("parkinson"[All Fields] AND "disease"[All Fields]) OR "parkinson disease"[All Fields] OR ("parkinsons"[All Fields] AND "disease"[All Fields]) OR "parkinsons disease"[All Fields]) OR ("spinal cord injuries"[MeSH Terms] OR ("spinal"[All Fields] AND "cord"[All Fields] AND "injuries"[All Fields]) OR "spinal cord injuries"[All Fields] OR ("spinal"[All Fields] AND "cord"[All Fields] AND "injury"[All Fields]) OR "spinal cord injury"[All Fields]) OR ("multiple sclerosis"[MeSH Terms] OR ("multiple"[All Fields] AND "sclerosis"[All Fields]) OR "multiple sclerosis"[All Fields]) OR ("amyotrophic lateral sclerosis"[MeSH Terms] OR ("amyotrophic"[All Fields] AND "lateral"[All Fields] AND "sclerosis"[All Fields]) OR "amyotrophic lateral sclerosis"[All Fields])) AND ("mesenchymal stem cells"[MeSH Terms] OR ("mesenchymal"[All Fields] AND "stem"[All Fields] AND "cells"[All Fields]) OR "mesenchymal stem cells"[All Fields])

**(3597 results)**
